# The onset of grapevine berry ripening is characterized by ROS accumulation and lipoxygenase-mediated membrane peroxidation in the skin

**DOI:** 10.1186/1471-2229-14-87

**Published:** 2014-04-02

**Authors:** Stefania Pilati, Daniele Brazzale, Graziano Guella, Alberto Milli, Cristina Ruberti, Franco Biasioli, Michela Zottini, Claudio Moser

**Affiliations:** 1 Research and Innovation Centre, Fondazione Edmund Mach, via E. Mach 1, 38010 San Michele a/Adige, TN, Italy; 2 Department of Physics, Bioorganic Chemistry Lab, University of Trento, Via Sommarive 14, 38123 Trento, Povo, Italy; 3 CNR, Istituto di Biofisica Trento, Via alla Cascata 56/C, 38123 Trento, Povo, Italy; 4 Department of Biology, University of Padova, Via U. Bassi 58/b, 35131 Padova, Italy

**Keywords:** Chloroplastic lipoxygenase, Fruit ripening, Galactolipids, Hydrogen peroxide, Oxidative stress, Oxylipin, ROS, Singlet oxygen

## Abstract

**Background:**

The ripening of fleshy fruits is a complex developmental program characterized by extensive transcriptomic and metabolic remodeling in the pericarp tissues (pulp and skin) making unripe green fruits soft, tasteful and colored. The onset of ripening is regulated by a plethora of endogenous signals tuned to external stimuli. In grapevine and tomato, which are classified as non-climacteric and climacteric species respectively, the accumulation of hydrogen peroxide (H_2_O_2_) and extensive modulation of reactive oxygen species (ROS) scavenging enzymes at the onset of ripening has been reported, suggesting that ROS could participate to the regulatory network of fruit development. In order to investigate this hypothesis, a comprehensive biochemical study of the oxidative events occurring at the beginning of ripening in *Vitis vinifera* cv. Pinot Noir has been undertaken.

**Results:**

ROS-specific staining allowed to visualize not only H_2_O_2_ but also singlet oxygen (^1^O_2_) in berry skin cells just before color change in distinct subcellular locations, i.e. cytosol and plastids. H_2_O_2_ peak in sample skins at véraison was confirmed by *in vitro* quantification and was supported by the concomitant increase of catalase activity. Membrane peroxidation was also observed by HPLC-MS on galactolipid species at véraison. Mono- and digalactosyl diacylglycerols were found peroxidized on one or both α-linolenic fatty acid chains, with a 13(S) absolute configuration implying the action of a specific enzyme. A lipoxygenase (PnLOXA), expressed at véraison and localizing inside the chloroplasts, was indeed able to catalyze membrane galactolipid peroxidation when overexpressed in tobacco leaves.

**Conclusions:**

The present work demonstrates the controlled, harmless accumulation of specific ROS in distinct cellular compartments, i.e. cytosol and chloroplasts, at a definite developmental stage, the onset of grape berry ripening. These features strongly candidate ROS as cellular signals in fruit ripening and encourage further studies to identify downstream elements of this cascade. This paper also reports the transient galactolipid peroxidation carried out by a véraison-specific chloroplastic lipoxygenase. The function of peroxidized membranes, likely distinct from that of free fatty acids due to their structural role and tight interaction with photosynthesis protein complexes, has to be ascertained.

## Background

Grapevine is an economically important crop, producing fruits that are consumed as fresh berries, pressed juice, dried berries and processed to make wine. Berry quality is determined by parameters measured at harvest, such as sugar content, acidity, skin color, berry size and polyphenol content. These depend on metabolic processes activated in the berry pericarp (skin and pulp) at the onset of ripening, reflecting a deep re-programming of the transcriptome [1-6]. Moreover, skin and pulp develop specialized features during ripening. In particular, skin accumulates anthocyanin to attract animals for seed-dispersal, provides a physical barrier against pathogens, avoid berry withering by preventing water loss and protects from solar radiation. This functional specialization is regulated at the transcriptional level [7]. Berry ripening inception is triggered by internal and external stimuli, via complex signal transduction pathways. Internal factors are hormones, such as auxins [8], abscisic acid [9,10], brassinosteroids [11] and ethylene [12,13]; metabolic factors, such sugar accumulation [9] and the increase of turgor pressure [14] and small signaling mediators, such as Ca^2+^[2,15]. An oxidative burst coinciding with berry color change and the modulation of reactive oxygen species (ROS) scavengers at the gene and protein level have been reported in grapevine, raising the possibility of ROS taking part to the signaling mechanisms occurring at fruit ripening [3,6,7,16,17].

Intracellular ROS can be generated by the incomplete reduction of oxygen or by energy transfer to an oxygen molecule. The first group of ROS are usually by-products of oxidative metabolisms such as respiration, photosynthesis and fatty acid oxidation, respectively occurring in mitochondria, chloroplasts and peroxisomes, and rapidly interconvert into the more stable hydrogen peroxide (H_2_O_2_). The latter is represented by singlet oxygen (^1^O_2_) and is produced by energy transfer at the phtosystem II reaction center, inside the chloroplasts [18]. Nonetheless, H_2_O_2_ can also be generated enzymatically by a family of NADPH-oxidases [19,20]. Despite their toxicity, at low levels ROS act as signaling molecules [21,22]. The specificity and selectivity of ROS signaling depend on the origin, reactivity and spatio-temporal accumulation of each ROS, as highlighted by a meta-analysis of ROS-related microarray experiments [23]. H_2_O_2_ is a signaling factor in plant response to external biotic and abiotic stimuli as well as in developmentally regulated processes (reviewed in [24]). H_2_O_2_ accumulation has been detected in numerous transitional phases of development: in grapevine at the moment of bud break [25], in sunflower during seed dormancy release [26], in tomato and grapevine at fruit ripening [3,27] and in Arabidopsis at floral transition [28]. ^1^O_2_ is the principal ROS that accumulates in illuminated photosynthetic tissues [29] and can trigger either acclimation or programmed cell death depending on the intracellular abundance [30,31]. A mechanism for plastid-to-nucleus ^1^O_2_ signaling is based on the generation of small volatiles derived from carotene oxidation which regulate transcription [32].

Among the most abundant molecules prone to ROS-induced damage, there are poly-unsaturated fatty acids (PUFAs), such as linolenic (18:3) and linoleic (18:2) acid. They can be oxidized by different molecules through different mechanisms generating specific regio- and stereo-isomers and this feature allows to identify *a posteriori* the ROS which accumulated. Indeed, lipid peroxidation can be generated either by nucleophilic attack of oxygen radicals, ^1^O_2_ direct addition or lipoxygenase and α-dioxygenase-catalyzed O_2_ addition [33]. Peroxidized fatty acid chains are rapidly converted into lower-molecular-weight compounds known as oxylipins [34,35], which can act as signaling molecules or be precursors of aromatic volatiles [36]. Jasmonic acid is an oxylipin derived via the lipoxygenase-mediated peroxidation of linolenic acid in the plastids, but also other oxylipins are known to play signaling roles in development [37] and defense [38].

Plant lipoxygenases (LOXs) are 95–100 kDa monomeric proteins with an N-terminal β-barrel domain (25–30 kDa), known as PLAT, probably involved in membrane or protein interactions, and a C-terminal α-helix-rich domain (55–65 kDa) containing the catalytic site, including a non-heme iron coordinated by five amino acid side chains and a water or hydroxide ligand [39]. They are classified according to the positional specificity of linoleic acid oxygenation, i.e. at carbon atom 9 (9-LOX) or 13 (13-LOX), leading to the formation of 9-hydroperoxy and 13-hydroperoxy derivatives (HpODEs and HpOT*r*Es). All plastidial LOXs are 13-LOXs and usually have a neutral pH optimum, whereas extra-plastidial LOXs can be either 9-LOXs or 13-LOXs and usually have an alkaline pH optimum [39].

We carried out a comprehensive analysis of the oxidative burst occurring in Pinot Noir grape berry skin at the onset of ripening to determine the potential signaling roles of ROS in fruit development. We also identified a plastidial LOX, likely responsible for galactolipid peroxidation and oxylipin synthesis, which might represent a novel component of this regulatory network.

## Results

### Singlet oxygen and hydrogen peroxide accumulate in Pinot Noir berry skin at the beginning of ripening

**Figure 1:**
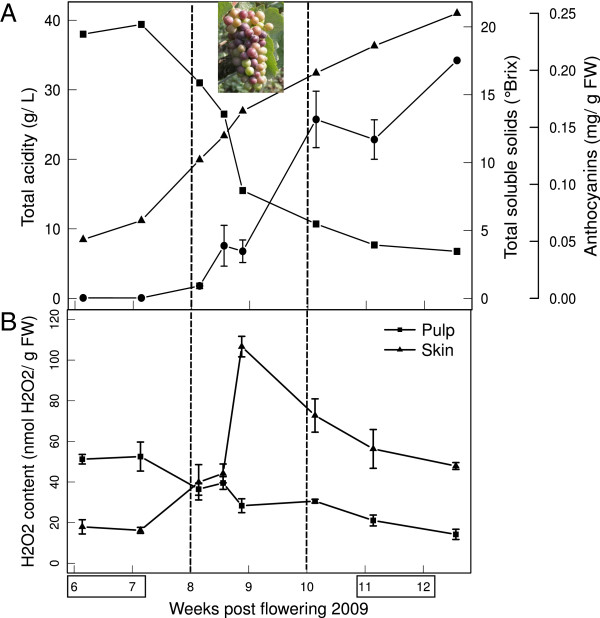
**H**_**2**_**O**_**2**_** content and biochemical changes in Pinot Noir berries during development. A**: Mean values of total acids (squares, expressed as grams of tartaric acid per liter) and sugars (triangles, expressed as total soluble solids in °Brix) of the must obtained from three clusters, at each time point. Berry skins anthocyanin content (circles) is expressed as grams of pelargonidin-3-glucoside per gram of berry fresh weight. **B**: H_2_O_2_ was measured separately in skin and pulp tissues of sampled berries. Data are means of three biological replicates ± se. The x-axis represents time in weeks post flowering (wpf). Véraison is indicated between dashed lines (8-10 wpf). Pre- (6-7 wpf) and post-véraison (11-12 wpf) stages are indicated by boxes. The picture of a cluster at mid-véraison shows the typical asynchrony of berries at this developmental transition.

The ripening of grapevine cv. Pinot Noir berries was followed during seven weeks starting from pre-véraison stage until mid-ripening (Figure 1A). Berries at pre-véraison (collected at 6 and 7 weeks post flowering (wpf)) were green and hard and were characterized by high content of organic acids and low content of sugars whereas berries sampled after 10 wpf were colored, soft, rich in sugars and with a low acidic content. The period between 8 and 10 wpf, named véraison, represents the transition to ripening during which crucial events occur: dramatic opposing changes of organic acids and sugars contents in the pulp, softening of the fruit and coloring of the skin. These changes do not take place in a synchronous way among berries of the same cluster, as shown in the picture of Figure 1A. As clusters were sampled by date and berries randomly pooled for must and pigment analyses, the obtained profiles, reported in Figure 1A, were smooth and diluted in time. Conversely, when sampling is based on physico-chemical characteristics of the berries, as for instance in [7], the differences between developmental stages are more sharp and larger.

H_2_O_2_ levels were measured separately in the skin and pulp of Pinot Noir berry samples (Figure 1B). While in the pulp a gradual decrease of H_2_O_2_ was observed, in the skin there was a clear accumulation of H_2_O_2_ at the beginning of ripening, with a maximum in samples collected at 9 wpf. This result leads to the conclusion that the transient peak in H_2_O_2_ content previously observed in whole berries at véraison [3] was actually contributed predominantly by the skin. A similar profile was observed in Pinot Noir during season 2008 (Additional file 1A). Taken in consideration the fact that samples collected by date are quite heterogeneous and that H_2_O_2_ accumulation is usually a fast event, its increase between 8 and 10 wpf likely corresponds to the proportion of berries undergoing the transition to ripening rather than to H_2_O_2_ increase within a single berry. As our interest is focused on cell signals, we did not further investigate the decreasing profile of H_2_O_2_ in the pulp, instead we characterized the events occurring in the skin.

### Imaging of ^1^O_2_ and H_2_O_2_ at the onset of ripening

**Figure 2:**
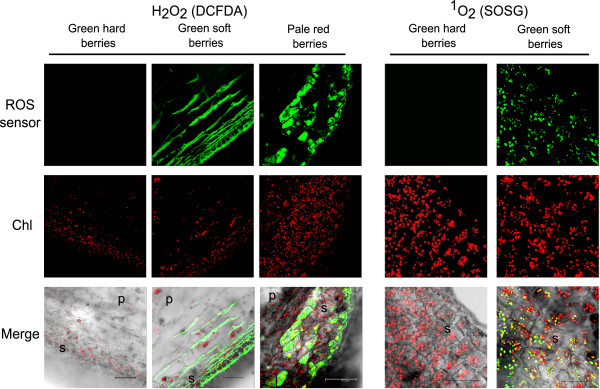
**Confocal images of Pinot Noir berries (100-μm sections) sampled at the green hard, green soft and pale red stages, stained for H**_**2**_**O**_**2**_**and**^**1**^**O**_**2**_**.** The sections were incubated with either 30 μM DCFDA or 30 μM SOSG (ROS sensors). Chlorophyll fluorescence has been recorded (Chl) to localize chloroplasts inside the cells. Merge is the computed overlay of the two fluorescence images and the bright field. Reference bars are 75 μm for H_2_O_2_ imaging and 25 μm for ^1^O_2_. Skin and pulp are indicated in the merge pictures with a “s” and “p”, respectively. For ^1^O_2_ imaging, only skin is visualized, at a higher magnification.

Single berries at the three developmental stages around the onset of ripening (green hard, green soft and pale red) were collected at 9 wpf in 2011 and sliced with a microtome to be used for ROS detection. ROS imaging was carried out by staining with three fluorescent dyes each specific for three type of ROS: dichlorofluorescein diacetate (DCFDA), which is sensitive to most ROS, hydroxyphenyl fluorescein (HPF), which is specific for strong oxidants such as the hydroxyl radical and peroxynitrite anion, and singlet oxygen sensor green (SOSG), which is specific for ^1^O_2_. Confocal images of sections stained with DCFDA and SOSG revealed the presence of ROS at the green soft and pale red stages and in the outer cell layers, i.e. those composing the skin (Figure 2, upper row). HPF did not yield a signal (not shown), suggesting that the ROS detected with DCFDA were weak oxidants, such as H_2_O_2_. The merge of the pictures obtained recording DCFDA/SOSG and chlorophylls fluorescence signals superimposed to the bright field showed that the localization of H_2_O_2_ and ^1^O_2_ was different at the subcellular level: H_2_O_2_ was detected in the cytosol whereas ^1^O_2_ exclusively in the plastids (Figure 2, bottom row).

### Catalase activity is strongly enhanced in the berry skin during ripening

**Figure 3:**
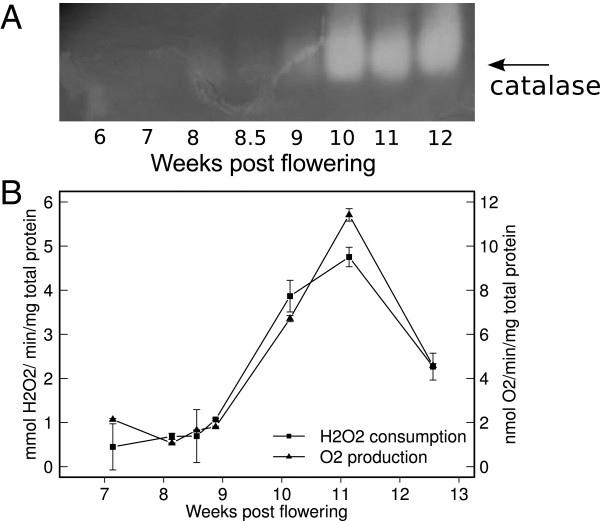
**Catalase activity during Pinot Noir berry development.** Native protein lysates were obtained from berry skins sampled at the indicated time points. **A**: Zymogram of catalase activity using 50 μg total proteins per lane. **B**: Catalase specific activity measured *in vitro* by determining either H_2_O_2_ consumption (absorbance at 240 nm) or O_2_ production (in-line O_2_ recording using direct injection MS). Data are means of biological duplicates ± se.

Catalase activity was investigated due to its relevance to H_2_O_2_ scavenging. It was initially visualized in total protein berry skin extracts by zymography as a strong single band in the samples collected at 10–12 wpf, indicating the activation of one specific isoform (Figure 3A). Catalase activity was then quantified *in vitro* by spectrophotometry (to measure H_2_O_2_ consumption) and by proton transfer reaction-mass spectrometry (to measure in-line O_2_ production), to unequivocally distinguish catalase from other scavenger activities (Figure 3B). Both assays confirmed the strong increase at 10 wpf, suggesting that catalase contributes to H_2_O_2_ scavenging after véraison. According to our results, the low level of H_2_O_2_ at pre-véraison cannot be attributed to a catalase scavenging activity and the following increase at véraison must thus be linked to an augmented ROS production, as commented in the discussion.

### Galactolipid peroxidation occurs at the onset of ripening

**Figure 4:**
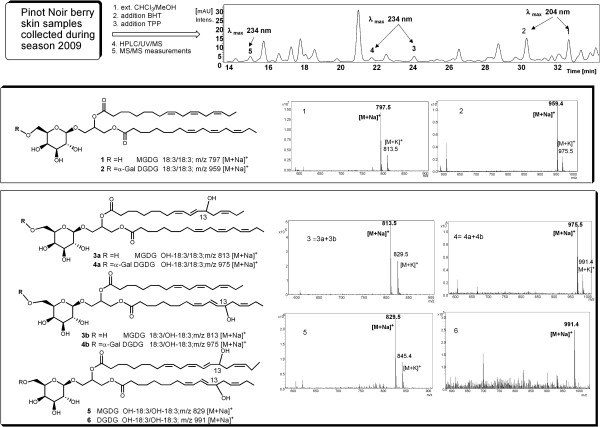
**Overview of the characterization study of galactolipids extracted from Pinot Noir berry skins at véraison (9 wpf).** The chromatogram shows eluted peaks recorded at 210 nm, with retention time shown on the x-axis. The mass spectra of the indicated peaks revealed that peaks 1 and 2 are attributable to MGDG 36:6 and DGDG 36:6. Peaks 3 and 4 are attributable to the corresponding mono-oxidized forms and peak 5 to the di-oxidized MGDG 36:6. Di-oxidized DGDG 36:6 has been identified but was barely detectable in the chromatogram.

Membrane lipids were analyzed with the aim to detect characteristic modifications caused by ROS accumulation. Crude lipid extracts were analyzed without pre-processing (e.g. fatty acid hydrolysis or derivatization) in order to study cell membrane lipid composition. Initially, the presence of peroxidized galactolipids at véraison was detected by MALDI-TOF mass spectrometry on extracts of berries collected during 2008 (Additional file 1B). Then, lipid extracts prepared from berries collected during 2009 season were analyzed by chromatographic separation coupled to mass spectrometry identification, as outlined in Figure 4. Three peaks absorbing at 234 nm were identified as oxidized lipids, as this wavelength is specific of the conjugated diene bonds formed during PUFAs oxidation. They were identified as the oxidized forms of monogalactosyl diacylglycerol and digalactosyl diacylglycerol carrying two α-linolenic fatty acid chains (MGDG 36:6 and DGDG 36:6). MGDG 36:6 and DGDG 36:6 were indeed the most abundant galactolipid species. Their structures were determined by full-scan electrospray ionization (ESI) in positive-ion mode (Figure 4, MS peaks 1 and 2) where they appeared as [M + Na]^+^ and [M + K]^+^ ion adducts and showed the same ion fragment at *m/z* 595 reflecting the loss of the corresponding sugar moiety. ESI-MS/MS on the [M + Na]^+^ ion adducts revealed strong fragment signals at *m/z* 519 (MGDG) and 681 (DGDG), reflecting the loss of linolenic acid at the primary position on the glycerol backbone, thus suggesting the presence of two identical 18:3 acyl chains in both the membrane lipids [40]. The analysis of purified samples containing MGDG and DGDG by ^1^H-NMR spectroscopy confirmed the presence of characteristic signals representing monogalactose (δ_H_ 4.23 d, 7.3 Hz for the α-acetal proton of β-galactose) and digalactose (δ_H_ 4.87 d, 3.7 Hz for the β-acetal proton of the α-galactose moiety in the digalactose structure) and also confirmed the presence of the 9Z,12Z,15Z octadecatrienoic (α-linolenic) acyl group for both the unsaturated chains.

Comprehensive HPLC-MS analysis of the peaks with lower retention times indicated the presence of more polar lipids in the extracts, strongly absorbing at 234 nm. These species gave ESI(+) mass spectra with ion adducts and fragment ions 16 Da heavier than the corresponding native galactolipids, indicating the presence of an additional hydroxyl group on one of the acyl chains (Figure 4, peaks 3 and 4). In the ESI(+) mass spectrum of peak 3 (λ_max_ 234 nm), the ions at *m/z* 813 and 611 therefore represent the mono-oxidized forms of MGDG 18:3/18:3 (peak 1, *m/z* 797 and 595), whereas in the ESI(+) mass spectrum of peak 4 (λ_max_ 234 nm), the ions at *m/z* 975 and 611 represent the mono-oxidized forms of DGDG 18:3/18:3 (peak 2, *m/z* 959 and 595). At lower retention times, we also detected di-oxidized forms of MGDG 18:3/18:3 (peak 5, [M + Na]^+^ at *m/z* 829, λ_max_ 234 nm) and DGDG 18:3/18:3 (peak 6, not showed in the chromatogram of Figure 4, [M + Na]^+^ at *m/z* 991, λ_max_ 234 nm). ESI-MS/MS of the mono-oxidized MGDG 18:3/18:3 (*m/z* 813) revealed two fragment ions at *m/z* 535 and 519 due to the loss of α-linolenic acid and oxidized α-linolenic acid, respectively. Because this neutral loss should occur more frequently at the primary glycerol position [40], the finding of equally populated fragment ions strongly indicates that the two acyl chains have a similar oxidation propensity. ESI-MS/MS of the di-oxidized MGDG 18:3/18:3 (*m/z* 829) revealed only one fragmentation at *m/z* 535 reflecting the loss of mono-oxidized α-linolenic acid, thus ruling out the presence of di-oxidized acyl chains.

**Figure 5:**
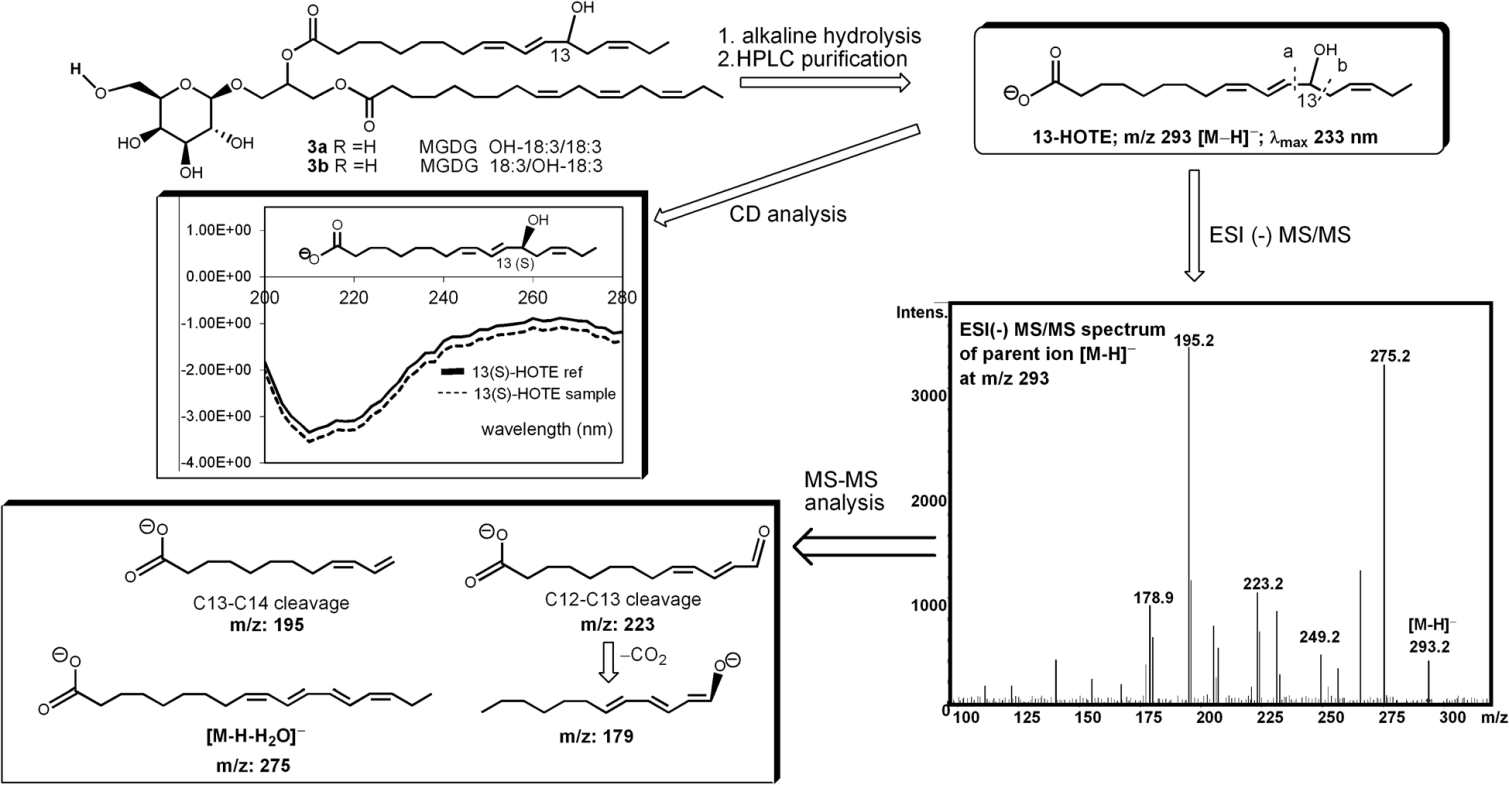
**Overview of the characterization study of the oxidized fatty acid chains obtained after hydrolysis of oxidized MGDG 36:6.** ESI MS/MS has been performed to assess the regiospecificity of the oxidation event and CD analysis has been performed to define its stereospecificity.

The regio and stereo-specificity of the hydroxyl group on the α-linolenic chain, obtained by alkaline hydrolysis of the oxidized MGDG 36:6, was then studied (Figure 5). Because fragmentation, besides common loss of neutral molecules (H_2_O and CO_2_), mainly occurs at the two C-C bonds adjacent to the carbon atom bearing the hydroxyl group, the intense daughter ions at m/z 195 and 223 obtained by collision-induced dissociation of the parent ion at m/z 293 (mono-oxidized α-linolenic carboxylate) unambiguously established the regiochemical position of the –OH function at the position 13 of the linolenic acyl chain [33]. Finally, we used circular dichroism (CD) spectroscopy to determine the absolute configuration of the C(13)-oxidized galactolipids. We found that the CD spectrum of the compound obtained after alkaline hydrolysis of the oxidized MGDG 36:6 from berry skins was identical to the CD spectrum of commercially available (9Z,11E,15Z)-*13-(S)*-hydroxyoctadecatrienoic acid (13HOT*r*E), thus indicating a 13-S absolute stereochemistry (Figure 5).

**Figure 6:**
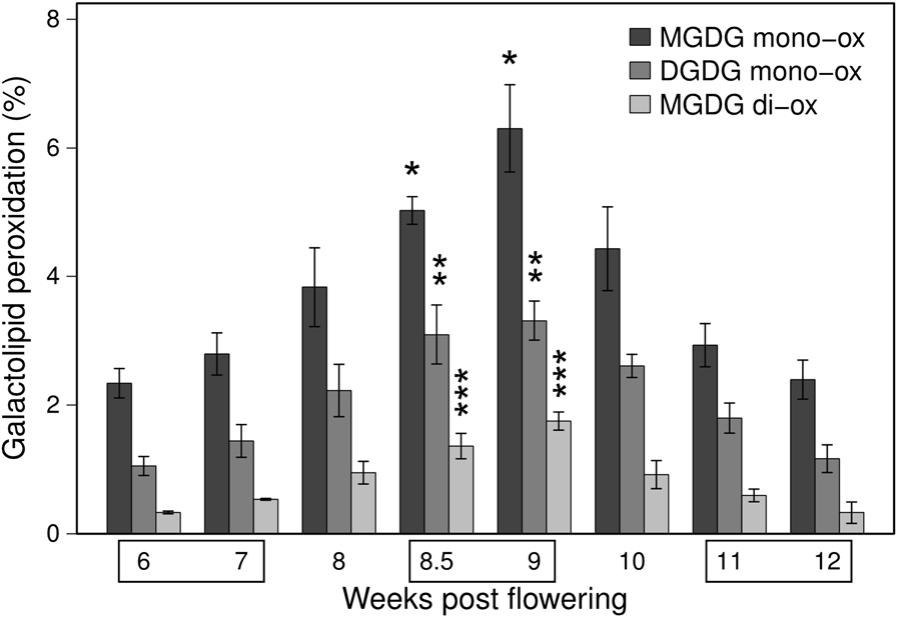
**Galactolipid peroxidation profiles during Pinot Noir berry development.** The mono-oxidized and di-oxidized forms of MGDG 36:6 and DGDG 36:6 are shown as percentage of total MGDG and DGDG, respectively. Data are means of three biological replicates ± sd. Lipid peroxidation at pre-véraison (6 and 7 wpf), véraison (8.5 and 9 wpf) and ripening (11 and 12 wpf) were analyzed by ANOVA and Tukey’s HSD (honestly significant difference) test. Asterisks indicate that the amount of peroxidized species accumulated at véraison is significantly different from that of the other two moments (p < 0.01).

Quantification of the oxidized MGDG and DGDG species in Pinot Noir berry skin along development showed a transient peak of accumulation at 9 wpf, mirroring the accumulation of H_2_O_2_ (Figure 6). By statistically comparing the relative amount of oxidized lipids present in the samples representing pre-véraison (6-7 wpf), véraison (8.5-9 wpf) and ripening (11-12 wpf) stages, it was evident that galactolipids oxidation state at véraison was significantly different from the other two stages considered. As the MGDG:DGDG ratio ranged from 1 to 0.8, the fact that MGDG reached a higher level of peroxidation (6% and nearly 2% for the mono- and di-oxidized forms vs. 3.5% of mono-oxidized DGDG) suggests that MGDG is oxidized preferentially. Moreover, even if di-oxidized MGDG showed the highest increase in terms of fold change, they accumulated to a lower extent than the mono-oxidized ones, suggesting they are less stable.

### A plastidial 13-lipoxygenase catalyzes galactolipid peroxidation at the onset of ripening

**Figure 7:**
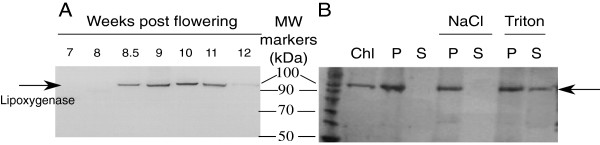
**Western blot analysis of lipoxygenase expression in Pinot Noir berry skin extracts. A**: Analysis of plastid lipoxygenases expression during berry development using a commercial antibody against Arabidopsis LOX2 and 10 μg of total protein extracts per lane. **B**: Analysis of plastid lipoxygenase expression in chloroplast-enriched samples obtained from fresh berry skins collected at 9 wpf. Total chloroplast protein extract (Chl) was fractionated into membrane (P) and soluble (S) fractions by centrifugation. Membrane pellets were treated with 1 M NaCl or 0.05% Triton X-100, incubated for 10 min on ice and centrifuged again to separate the membrane (P) and the soluble (S) fractions. Pellets were resuspended in a volume identical to the corresponding soluble fractions and loaded in equal amounts for separation by SDS-PAGE and detection by western blot. MW markers: molecular weight markers (kDa).

Western blot analysis of total protein extracts obtained from berry skin samples collected during 2009 was performed using a commercial antibody raised against the Arabidopsis plastidial LOX2 to characterize the presence of LOX activity in concurrence with galactolipid peroxidation. A single 95–100 kDa band was observed in the samples harvested from 8.5 to 11 wpf (Figure 7A). We wanted to identify the proteins contained in that band by MS analysis, but their amount was below the instrument sensitivity. In the attempt to enrich the sample in chloroplastic LOXs, plastids were isolated from fresh berry skin collected in 2011 at the green soft/pink stage (9 wpf) using a Percoll gradient.Chloroplasts were lysed and their content partitioned into stromal and thylakoid-enriched fractions. All the obtained fractions were analyzed for LOX expression by western blot (Figure 7B). Pinot Noir LOX was found predominantly in the thylakoid-enriched fraction, which was then used for tryptic digestion and MS analyses (Additional file 2). nanoLC/MS sequencing identified one peptide unambiguously matching Vv06s0004g01510, a 13-LOX differing at only five out of 901 residues from the recently described Sauvignon Blanc LOXA [41]. This result was confirmed by comparative MALDI-TOF/MS analysis performed on this fraction and on recombinant Vv06s0004g01510 protein. We therefore named the protein PnLOXA.

**Figure 8:**
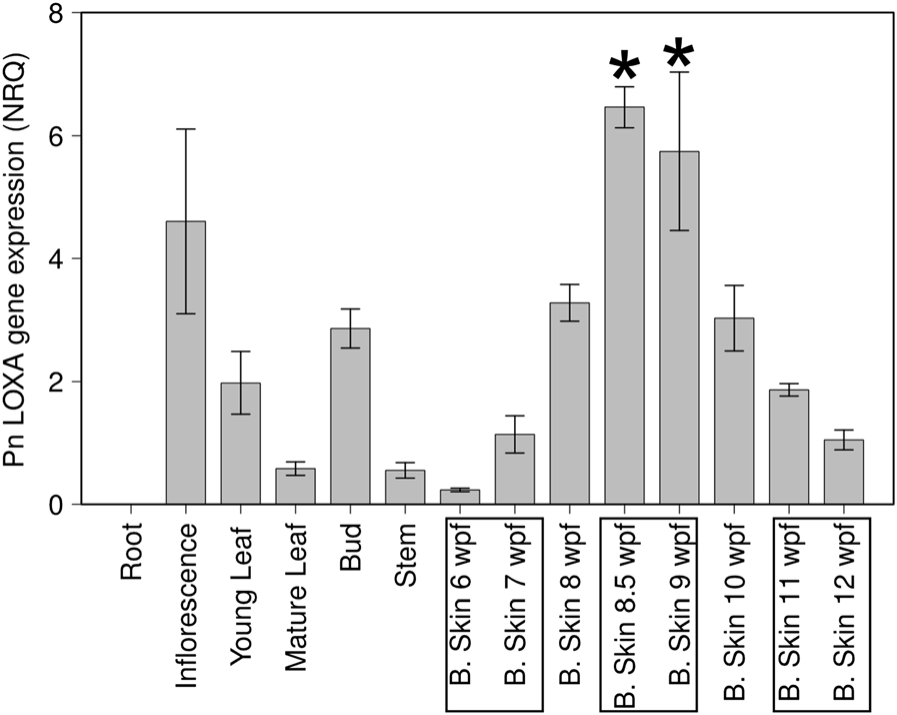
***PnLOXA *gene expression in grapevine tissues and in berry skins along development (6–12 wpf).** Normalized relative quantities ± se were calculated using three reference genes; *n* = 3. *PnLOXA* expression at véraison (marked by asterisks) was significantly different from pre-véraison (6-7 wpf) and ripening (11-12 wpf) as assessed by ANOVA and Tukey HSD test (p < 0.01).

**Figure 9:**
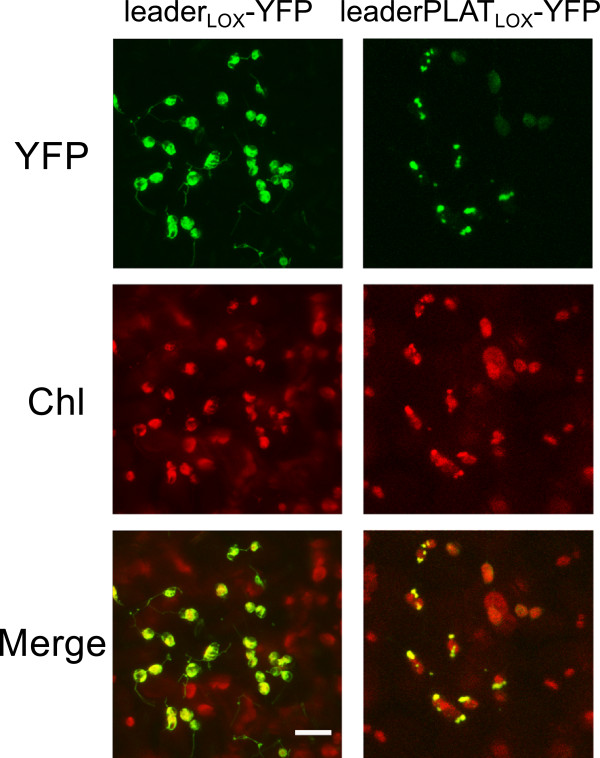
**PnLOXA localization demonstrated by the expression of YFP fusion constructs in grapevine leaves.** Leaves were infiltrated with *Agrobacterium tumefaciens* carrying the pGreen[Pn*LOXA*transitpeptide_1-47_-YFP] and pGreen[Pn*LOXA*transitpeptidePLAT_1-220_-YFP] constructs. Chlorophyll (Chl) and YFP fluorescence were recorded using Leica SP II confocal microscope. Merge is the computed overlay of the two fluorescence images. Reference bar is 10 μm.

*PnLOXA* gene expression was analyzed by RT-PCR in a panel of Pinot Noir tissues and in developing berry skin (season 2009). We observed a 20-fold increase in its expression at the onset of ripening (Figure 8) matching precisely with the peaks of protein abundance detected by western analysis (Figure 7A) and of galactolipid peroxidation (Figure 6). Statistical comparison among the three berry development stages defined above highlighted that *PnLOXA* expression at véraison was significantly different from pre-véraison and ripening stages. *PnLOXA* expression was not restricted to the berry. Indeed, the gene was expressed in all the photosynthetic tissues we analyzed, particularly in plant structures undergoing developmental changes (such as bud and inflorescence). These results agree with *in silico* analysis of *LOX* gene expression in the grapevine atlas ([42], Additional file 3A): the only tissues where *PnLOXA* is not expressed are woody stem, root and senescent leaf while in winter bud it is minimally expressed. Conversely, it is highly expressed in inflorescence, flower, bud, tendril and berry at véraison. The atlas data show that five *LOX* genes are modulated during berry development: two *9-LOX* (Vv05s0020g03170 and Vv14s0128g00790) and three *13-LOX* genes (*PnLOXA*, *Vv09s0002g01080* and *Vv01s0010g02750*). However, only *PnLOXA* shows an induction at véraison (Additional file 3B). Primary structure analysis of PnLOXA indicated the presence of a plastid targeting peptide (residues 1–47), a PLAT domain which might be involved in protein-protein or protein-lipid interactions (72–204), and a C-terminal catalytic domain that coordinates Fe^3+^ (207–901). We created two fusion constructs with yellow fluorescent protein (YFP): one containing only the transit peptide to study PnLOXA intracellular localization and the other containing also the PLAT domain to gain insights into its function. Transient expression of the first construct in grapevine and tobacco leaves followed by confocal imaging showed that YFP was efficiently translocated into the chloroplasts (Figure 9 and Additional file 4, left column). The presence of the PLAT domain is responsible of a non-uniform distribution of YFP fluorescence inside the plastid, consistent with that of a thylakoid-associated protein (Figure 9 and Additional file 4, right column). Similar results, showing a spot-like localization inside the chloroplast, were reported for potato and tomato lox [43,44]. The *in vivo* localization supports the previous chloroplast fractionation experiment (Figure 7B) and suggests that the PLAT domain is involved in protein localization at the thylakoid.

**Figure 10:**
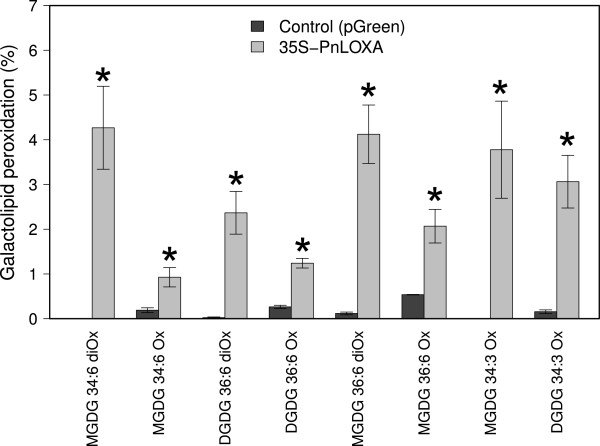
**Galactolipid analysis of tobacco leaves transiently expressing *PnLOXA*.** Leaves transformed either with the *PnLOXA* or the empty vector (pGreen) as control were collected 7 days after Agrobacterium inoculation. Galactolipid peroxidation is reported as a percentage of mono- and di-oxidized species within each class, normalized on the amount of PnLOXA protein. Data are means of three replicates ± sd. ANOVA and Tukey HSD test were performed to compare control and *PnLOXA* over-expressing samples. Asterisks indicate significant differences from control at p < -0.05.

Finally, we analyzed PnLOXA enzymatic activity to confirm its ability to peroxidize free fatty acid chains and also membrane galactolipids. The mature protein was firstly expressed in *E. coli*, purified by ion-chelating affinity chromatography and tested *in vitro*. PnLOXA catalyzed the regiospecific peroxidation of α-linolenic acid to produce exclusively 13-HOT*r*E (ESI-MS/MS analysis). To test the ability of the enzyme to catalyze the peroxidation of galactolipids, we incubated PnLOXA with the most pure galactolipid fraction isolated from grape berry skins, which was that enriched in DGDG. PnLOXA efficiently catalyzed the 13-peroxidation of DGDG 36:6, producing both mono-oxidized (3.6%) and di-oxidized products (5.6%). Table 1 shows the degree of peroxidation within each DGDG species: the prevalence of di-oxidized forms indicates that PnLOXA acts on both galactolipid chains without significant discrimination. We also studied membrane lipid peroxidation *in vivo* by transiently overexpressing *PnLOXA* in tobacco leaf cells. We agro-infiltrated leaves with either the *PnLOXA* construct or the empty vector and collected leaf transformed spots during the following days for protein expression analysis. Overexpression of *PnLOXA*, monitored by western blot, reached a maximum at 7 days after transformation (not shown). The experiment was then repeated in biological triplicates collecting samples 7 days after infiltration and lipid extracts were analyzed by HPLC-MS. The amount of oxidized species in the control samples was nearly detectable, whereas the presence of the grapevine enzyme caused a statistically significant increase of galactolipid peroxidation (Figure 10). The amount of peroxidized galactolipids was normalized to the amount of PnLOXA protein actually present in each replicate (see Additional file 5) and used to calculate the average peroxidation value. As in grapevine berry skin, also in tobacco leaves MGDG seem preferentially oxidized; however in the latter, di-oxidized galactolipids accumulate more than mono-oxidized species (as observed *in vitro*, Table 1).

**Table 1:** ***In vitro *** **galactolipids peroxidation after incubation with purified recombinant PnLOXA (10 minutes at 25°C), expressed as relative percentage over total DGDG within each class**

	**Chain composition**	**Mono-oxidized (%)**	**Di-oxidized (%)**	**Relative abundance in the extract (%)**
DGDG 36:6	18:3/18:3	1.6	9.8	51.3
DGDG 34:3	18:3/16:0	9.3	0	14.2
DGDG 36:3	18:3/18:0	9.3	0	13.9
DGDG 36:4	75% 18:3/18:1 25% 18:2/18:2	8.5	1.2	7.6
DGDG 36:5	18:2/18:3	0	7.6	6.4

## Discussion

The transition from mature green to ripening berries is a crucial developmental phase in grapevine, as well as in many fleshy fruits, because it involves broad metabolic reprogramming and definitive specialization. Internal signals (developmental, hormonal and metabolic) refined by external cues trigger a set of integrated regulatory cascades, possibly including a burst of oxidative stress, at the transition to the ripening phase [3,6,7,16,17]. This study definitely confirms the transient accumulation of H_2_O_2_ in the cytosol of berry skin cells at the beginning of ripening and shows the concomitant accumulation of ^1^O_2_ inside chloroplasts (Figure 2), where also enzymatic peroxidation of membrane galactolipids occurs.

Although it is difficult to measure H_2_O_2_ in plant tissues accurately [45], it clearly accumulates in berry skin at softening and color change (Figures 1, 2 and Additional file 1A). Basal levels are probably restored by the activity of a catalase isoform which is specifically expressed and active since 10 wpf (Figure 3). This catalase isoform resembles Arabidopsis CAT3, which is stress- and substrate-inducible and is expressed at bolting time, when a peak of H_2_O_2_ occurs in the leaves and senescence is triggered [46]. We have no evidence to attribute the accumulation of H_2_O_2_ to a down-regulation of scavenger activities, at least of catalase, rather we might speculate about an increase in ROS production at ripening onset. Potential sources could be chloroplasts, which are undergoing a transition to non-photosynthetic organelles, or mitochondria, which transiently shift to an aerobic fermentative metabolism [47].

H_2_O_2_ accumulation and catalase activity are reported also at bud-break in grapevine [48-51], where the role of H_2_O_2_ as a signal molecule in the release of buds endodormancy has been proposed.

In plants, ^1^O_2_ is usually generated at photosystem II by energy transfer from excited triplet chlorophylls to triplet oxygen (O_2_) under photo-oxidative conditions [52]. At the onset of ripening, a developmentally regulated switch off of photosynthesis occurs and ^1^O_2_ is likely to be generated. Quite unexpectedly, we do not detect significant oxidative damage on thylakoid membrane lipids attributable to ^1^O_2_, rather the lipoxygenase-mediated accumulation of 13-peroxy galactolipids (Figures 4 and 5). At 9 wpf, 6% of the MGDG and 3.5% of the DGDG are oxidized on one chain and nearly 2% of the MGDG are oxidized on both chains (Figure 6). A grapevine plastidial 13-lipoxygenase (PnLOXA) probably responsible for the transient galactolipid peroxidation in Pinot Noir grapes has been identified. It differs at only five out of 901 residues from the Sauvignon Blanc orthologue [41]. The véraison-specific expression profile of this LOX isoform (Figures 7 and 8) was already highlighted in a proteomic study which proposed it as a biomarker of grapevine ripening [53]. According to the Vitis atlas [42] other two 13-*LOX* genes are expressed in the berry, but with a descending profile from fruit set to full ripening. Moreover, one of these, *LOXO*, is induced by abiotic and biotic stresses, such as wounding and Botrytis infection [41] and is regulated by VvWRKY1 in response to downy mildew [54]. An important feature of PnLOXA is the ability to peroxidize membrane galactolipids both *in vitro* and *in vivo* (Table 1 and Figure 10) and not only free fatty acid chains, as it is usually assumed. Moreover, PnLOXA causes the preferential accumulation of di-oxidized forms of MGDG and DGDG. We can thus conclude that in the fruit skin the di-oxidized MGDG do not accumulate due to a very fast scavenging or conversion. Similar conclusions were reported for Arabidopsis chloroplastic lipoxygenase LOX2 [55]. The study of *lox2* mutant suggested that LOX2 could directly oxidize membrane galactolipids and that di-oxidized forms were strictly related to its presence, whereas mono-oxidized forms accumulation occurred also in a *lox2* background. Finally, the preferential accumulation of oxidized MGDG was observed: we speculate that this phenomenon could be related to a PLAT-mediated specific localization of PnLOXA at the thylakoid (Figure 9), rather than to substrate discrimination. In fact, MGDG and DGDG have distinct structural properties and distribution in the membrane and there are proteins known to interact preferentially with MGDG, such as violaxanthin de-epoxidase and cytochrome b6f [56,57].

The biological function of enzymatically generated membrane peroxy-lipids in the chloroplast at the onset of ripening is not clear yet. Usually peroxidation occurs on free fatty acid chains and generates, through catalyzed or spontaneous reactions, compounds called oxylipins, among which the hormone jasmonic acid [34]. The signaling function of oxylipins is well established, as many studies have demonstrated their influence on physiological processes such as root development and plant defense in Arabidopsis [37] and light acclimation in Chlamydomonas [58]. Besides, some oxylipins are volatile aromatic compounds, such as C6 volatile aldehydes, alcohols and esters, which confers the characteristic flavors to fruits including grapes and wine [36]. In tomato, a chloroplastic LOX expressed in the fruit at the moment of color change, named *TomLOXC* (U37839), has been related to the aroma flavor of ripe fruits [44,59-61]. A phylogenetic analysis based on protein sequence similarity shows that TomLOXC and PnLOXA belong to the same group of chloroplastic 13-LOX (Additional file 6), suggesting they could have conserved functions in the two fruits. The observation that their expression pattern is centered at véraison rather than at ripening, when the aroma are accumulated, and that *TomLOXC* is directly activated by the MADS box transcription factor RIN, which is a major regulator of the onset of ripening in tomato [62], strongly support the hypothesis of these LOXs participation to fruit development signaling. Moreover, the peculiarity of PnLOXA of peroxidizing membrane lipids instead of free fatty acid chains allows to speculate on at least other two possible functions of peroxy-lipids. On the one hand, membrane peroxidation could undergo fragmentation and generate a particular class of oxylipins, namely phytoprostanes with signaling function [35,63], while on the other hand it could regulate membrane proteins activity by reversible oxidation of active-site cysteines, as reported for a human protein tyrosine phosphatase [64].

## Conclusion

This work sheds light on the oxidative species transiently accumulating in the skin of grapevine berries at the onset of ripening. Skin cells are continuously exposed to solar radiation, even during the programmed dismantling of the photosynthetic apparatus at the onset of ripening. In this transitional phase, ROS could exceed skin cells scavenging capacity, accumulate and affect the transcription of nuclear genes involved in photo-protection and ROS-scavenging. Besides, the enzymatic peroxidation of thylacoidal membranes may represent the first step in oxylipin synthesis or a mechanism to regulate membrane proteins through redox control.

## Methods

### Plant material and biochemical analysis

Three clusters of grape berries (*Vitis vinifera* cv. Pinot Noir ENTAV115) were collected during 2008 and 2009 at FEM study site between 9 and 10 am at eight time-points between 6 and 13 weeks post flowering (wpf, flowering is intended as 50% of open flowers in the vineyard). Half of each cluster was immediately frozen in liquid nitrogen and the other half was pressed for must analysis by means of Fourier transform infrared spectroscopy (FTIR) using the instrument WineScanTM Type 77310 (Foss Electric, Denmark). Frozen berries were peeled with a scalpel, separating the most external cell layers (exocarp, skin) from the rest (mesocarp and endocarp, pulp). Skin and pulp, were ground separately to obtain a fine powder. The anthocyanin concentration in the skin was measured after methanol extraction (1 g berry skin powder in 10 ml methanol) according to the double pH differential method [65]. H_2_O_2_ was measured using the Amplex UltraRed (Molecular Probes, USA) as described in [3] but using 10 μl of aqueous extracts instead of 50 μl.

For confocal microscopy experiments and plastid isolation, fresh berries were collected at the green soft and color change stages during 2011.

Total protein was extracted from 6 g of skin powder plus 25% (w/w) PVPP in 10 ml lysis buffer (0.2 M sodium phosphate buffer, pH 7.5, 5 mM EDTA) containing protease inhibitors (Sigma, MO). Samples were incubated on ice for 10 min then centrifuged for 15 min at 15,000 × *g* at 4°C. The supernatant was clarified by precipitation with 30% ammonium sulfate and centrifugation at 20,000 × *g* for 30 min, washed to remove salts and concentrated on Amicon Ultra (Millipore). The protein concentration was determined using QuantIt (Invitrogen).

*In vitro* catalase activity was measured by spectrophotometry and mass spectrometry. In the first assay, catalase activity was measured in 250 μl phosphate buffer (pH 7) at 25°C using 10 μg of grape skin protein extract following [66]. The specific catalase activity was calculated using the H_2_O_2_ molar extinction coefficient at 240 nm (43.6 M^-1^cm^-1^) and was expressed as moles of H_2_O_2_ consumed per min per mg total protein. In the second assay, catalase activity was measured in 180 ml 50 mM potassium phosphate buffer (pH 7) in the presence of 50 mM H_2_O_2_ with a continuous nitrogen flux taking volatiles to the PTR-TOF-MS (Ionicon Analytik, Austria). The production of O_2_ was measured using the signal at *m/z* 32 after the addition of 1 mg grape skin protein extract. Preliminary calibration using bovine catalase (Sigma-Aldrich) showed that maximum O_2_ production (O_2max_) was proportional to the enzyme concentration in the solution so O_2max_ was used to quantify catalase activity in the extracts.

Native gel and zymography catalase detection was done as described by [46]. SDS-PAGE was performed using pre-cast 4–12% NuPAGE gels and 1× MOPS running buffer (Invitrogen). Proteins were transferred onto PVDF membranes (Millipore) using an XCell II Blot Module (Invitrogen) at 40 V for 1 h. Membranes were blocked with 5% BSA in TBST buffer for 1 h at room temperature and incubated overnight at 4°C with the primary antibody. Anti LOX-C plastidial lipoxygenase (AS07258, Agrisera) was used to detect VvLOXs. Anti catalase (AS09501, Agrisera) was used to detect VvCATs. Alkaline phosphatase-conjugated goat anti-rabbit AffiniPure (Jackson ImmunoResearch) was used as the secondary antibody; detection was performed using Alkaline phosphatase blue membrane substrate solution (Sigma-Aldrich).

### Preparation of chloroplasts

Chloroplasts were prepared from 20 g skin tissue as described by [67]. Intact plastids were recovered using a 40–80% Percoll gradient, washed with hypotonic lysis buffer and centrifuged for 10 min at 10,000 × *g* to separate soluble and membrane-associated proteins. The pellet was divided into three parts, which were dissolved in 0.1% TritonX-100 or 1 M NaCl or control buffer. These three samples were centrifuged as above and the final soluble and membranous fractions were prepared for SDS-PAGE.

### Cloning and expression of recombinant *PnLOXA*

The *PnLOXA* coding sequence (Vv06s0004g01510) without the plastid targeting sequence was amplified from Pinot Noir berry cDNA using Phusion DNA polymerase (Finnzymes) and the primers LOXfw5′BamHI (5′-GGATCCGTTGGCTACGTCCCTG-3′) and LOXrev3′HindIII (5′-AAGCTTTCAAATGGAGATACTGTATGGAA-3′) and inserted into the pGEM-T vector for sequencing. *PnLOXA* was then transferred to the expression vector pQE30 using the BamHI/HindIII restriction sites, thus adding an N-terminal His_6_ tag. *Escherichia coli* M15 [pRep4] cells transformed with pQE30:*PnLOXA* were induced with 1 mM IPTG and 2% ethanol for 16 h at 20°C. A 1-L bacterial culture pellet was resuspended in 40 ml of 50 mM HEPES/NaOH buffer (pH 7.5) containing 150 mM NaCl, 5 mM DTT and protease inhibitors (Sigma). After sonication and lysozyme treatment (0.2 mg/ml), the cleared bacterial lysate was adjusted to 0.5M NaCl and loaded onto a 5-ml HisTrap™ FF crude Column (GE Healthcare, AKTA Purifier system) pre-equilibrated with binding buffer (20 mM HEPES-NaOH pH 7.5, 0.5 M NaCl). The column was washed with binding buffer and 50 mM imidazole (Merck), and the His_6_-PnLOXA protein was eluted using 250 mM imidazole. Protein yield was 2 mg of pure protein per liter of bacterial culture. Recombinant PnLOXA activity was studied using 0.1 mM α-linolenic acid (Sigma) at pH 6.5 and 25°C.

### Transient expression in grapevine and tobacco leaves

Three constructs were prepared for transient expression: two YFP fusion constructs for localization analysis, and the complete *PnLOX*A gene for functional analysis. The *YFP* coding sequence was subcloned from vector pAVA554-p35S-YFP [68] into pSAT1-p35S-nVenus [69] using the restriction enzymes NcoI and BglII. The p35S-YFP cassette was then inserted into pGreen0029 using the EcoRV/NotI sites [70]. The 1-47 and 1-220 *PnLOXA* peptides were amplified using primers 5′leaderBspHI (5′-TTGCTCATGATGTTCAAGACTCAGGTCCA-3′), 3′leaderBspHI (5′-GCAGTCATGAGGCCAACCCTAACATTCCT-3′) and 3′PLATBspHI (5′-CTTGATCATGACTGGTGTTTCCAATGGTAAGT-3′). The PCR products were digested with BspHI and inserted into pGreen0029-35S-YFP, digested with NcoI. The pGreen[Pn*LOXA*transitpeptide_1-47_-YFP] and pGreen[Pn*LOXA*transitpeptidePLAT_1-220_-YFP] binary vectors were introduced into the *Agrobacterium tumefaciens* strain GV3101-pSoup [69,71] as described by [72].

The complete *PnLOXA* coding sequence was amplified from Pinot Noir cDNA using primers 5′leaderBspHI and LOXrev3′HindIII. The amplified product was introduced into vector pUC19 and sequenced. The 2450 bp-NcoI/XbaI *PnLOXA* fragment from the pUC19-*PnLOXA* was cloned into the pGreen[*PnLOXA*transitpeptidePLAT_1-220_-YFP] vector previously digested with NcoI and XbaI, obtaining the pGreen[*PnLOXA*].

For *PnLOXA* transient expression in tobacco leaves, Agrobacterium transformed either with the pGreen empty vector or the pGreen[35S:*PnLOXA*] was inoculated into leaves of six tobacco plants, so that each tobacco plant was a biological replicate. For time-course expression analysis, leaves were collected from 4 to 12 days after infiltration and protein extracts analyzed by western blot. For galactolipid peroxidation analysis, western blot was used to quantify the PnLOXA expression in each biological replicate using Image J software and lipids were extracted as described below for HPLC-MS analysis.

### Lipid analysis

Total lipids were extracted from frozen Pinot Noir berry skin samples according to [73]. We added 1 mM 3,5-di-tert-butyl-4-hydroxytoluene (BHT) to the extraction buffer to prevent oxidation during sample preparation and 1 mM triphenylphosphine (TPP) to reduce hydroperoxyl groups to hydroxyl groups, which are more stable and suitable for quantitative analysis. The extracts were dissolved in 90:10 methanol/chloroform and 5 μL were injected into a Hewlett-Packard Model 1100 Series liquid chromatograph (Hewlett-Packard Development Company, CA) coupled to a photodiode array (PDA) detector (Agilent Technologies, Italy, Agilent 1100 Series) and to a Bruker Esquire-LC quadrupole ion-trap mass spectrometer (Bruker Optik GmbH, Germany) equipped with atmospheric pressure electrospray ion source. Analysis was carried out at room temperature on an Agilent ZORBAX Eclipse XDB-C8 150 × 4.6 mm, 3.5 μm column. The eluent (0.8 mL/min) consisted of (A) methanol: water/12 mM ammonium acetate (70:30) and (B) methanol/12 mM ammonium acetate using a linear gradient: 35%–100% B in 40 min, followed by isocratic B held for 10 min. The details of the MS parameters have been described previously [40,74]. The regiochemical distribution of galactolipids was established as described by [40] using either short-wavelength UV-DAD or ESI-MS detection. The relative percentage of peroxidation of MGDG 36:6 and DGDG 36:6 in all the samples was established by the ratio of the absolute ESI (+) area of the extracted ion current (EIC) of each oxidized product with respect to the ESI (+) area of the extracted ion current (EIC) of total (native and oxidized) MGDG and DGDG, respectively.

Galactolipids were purified by Si-60 flash chromatography and chloroform-methanol gradient elution. Near pure (TLC analysis) MGDGs and DGDGs were obtained in fractions 9 and 10, respectively. Fraction 10 was used as the DGDG substrate for *in vitro* enzymatic oxidation. Portions of fraction 9 and 10 were purified further by reverse phase HPLC (methanol/water gradient elution) to obtain pure (NMR analysis) MGDG 18:3/18:3 (1.5 mg) and DGDG 18:3/18:3 (1.4 mg).

MGDG 18:3/18:3 were hydrolyzed in methanol solution (1 mM, 200 μL) by treatment with an aqueous KOH solution (500 mM, 300 μL) for 1 h at room temperature. The basic solution was neutralized with 500 mM HCl, and an organic extract was obtained by extracting three times with 400 μL n-hexane. LC-MS analysis confirmed the presence in the hexane extracts of linolenic acid (~90%) and of its corresponding 13- hydroxy derivative (13-HOT*r*E). The peak corresponding to the latter was collected, evaporated and rinsed with 1 ml methanol for chiral analysis. The CD spectrum of methanolic 13*-*HOT*r*E was recorded with a Jasco J710 spectropolarimeter.

^1^H-NMR spectra were obtained for MGDG 18:3/18:3 and DGDG 18:3/18:3 by dissolving each in 600 μL tetradeuterated methanol (99.9% CD3OD, Aldrich) and carrying out measurements at 298 K on a Bruker-Avance 400 MHz spectrometer with a 5-mm BBI probe set at a 90° proton pulse length of 9.4 μs and a transmission power of 0 db. The chemical shift scale (δ) was calibrated on the residual proton signal of deuterated methanol at δ_H_ 3.310 ppm.

### Confocal imaging

ROS sensitive fluorescent dyes and YFP were imaged using a Leica SP5 confocal microscope (Leica, Germany). Dichlorofluorescein diacetate (DCFDA), hydroxyphenyl fluorescein (HPF) and singlet oxygen sensor green (SOSG, Molecular Probes) staining was carried out by preparing 100 μμm berry sections on a microtome, and incubating them for 2 h at room temperature in the dark with 30 μM DCFDA, 30 μM SOSG or 10 μm HPF dissolved in 50 mM sodium phosphate buffer (pH 7.5). The excitation wavelength was 488 nm and the emission wavelengths were 500–535 nm, 500–540 nm, 505–535 nm and 675–725 nm for DCFDA, SOSG, HPF and chlorophyll, respectively.

For PnLOXA localization study, leaf pieces from the agroinfiltrated areas collected at 6 days after infiltration were mounted on slides. YFP detection was carried out using excitation and emission wavelengths of 488 nm and 515/530 nm, respectively.

Confocal images were processed using ImageJ software.

### Quantitative RT-PCR

Total RNA was extracted from grapevine tissues using the Spectrum Total Plant RNA kit (Sigma) and quantified using Nanodrop 8000 (Thermo Scientific). Integrity was checked by capillary electrophoresis using Bioanalyzer 2100 (Agilent). First strand cDNA was synthesized from 2 μg RNA using the SuperScript VILO cDNA Synthesis Kit (Invitrogen) according to the manufacturer’s instructions, with the primers indicated in Additional file 7. The cDNAs were mixed with Fast SYBR Green Master Mix (Applied Biosystems) and amplified on a ViiA 7 Real Time PCR System (Applied Biosystems) using an initial heating step of 95°C for 20 sec, followed by 40 cycles of 95°C for 1 sec and 60°C for 20 sec. Raw fluorescence data were extracted using Viia 7 Software v1.0. Ct and reaction efficiency were calculated using LinRegPCR software [75]. Relative expression was calculated according to [76] by centering expression values for each gene on the mean value. Three reference genes out of five (ubiquitin, SAND and GAPDH) were selected by geNorm and used for normalization [77].

## Abbreviations

ROS: Reactive oxygen species; MGDG: Monogalactosyl diacylglycerol; DGDG: Digalactosyl diacylglycerol; H2O2: Hydrogen peroxide; 1O2: Singlet oxygen; 13HOTrE: (9Z,11E,15Z)-*13-(S)*-hydroxyoctadecatrienoic acid.

## Competing interests

The authors declare that they have no conflict of interest.

## Authors’ contribution

SP substantially contributed to conception, performed enzymatic assays, statistical analysis and manuscript drafting. DB carried out ROS visualization at confocal microscope and helped in biochemical assays. GG performed all lipid identification and analyses. AM identified the lipoxygenase protein by mass spectrometry. CR and MZ carried out the tobacco and grapevine transient expression and localization analyses. FB carried out catalase activity assay by PTR-MS. CM substantially contributed to the study conception and design and manuscript drafting. All authors read and approved the final manuscript.

## Supplementary Material

Additional file 1 **H**_**2**_**O**_**2**_** content quantification and lipid peroxidation analysis of Pinot noir berry skin extracts during season 2008.** Click here for file

Additional file 2 Mass spectrometry analyses results for PnLOXA identification. Click here for file

Additional file 3 **Updated description of the *Vitis vinifera LOX *** **gene family; heatmap of the *LOX *** **gene family expression in the *V. vinifera *** **cv Corvina atlas and transcriptional profiles of the 5 *LOX *** **genes expressed in Corvinia berries.** Click here for file

Additional file 4 PnLOXA localization demonstrated by transient expression of YFP fusion constructs in tobacco leaves. Click here for file

Additional file 5 **Western analysis of tobacco leaves transiently overexpressing *PnLOXA*.** Click here for file

Additional file 6 **Phylogenetic analysis of LOX sequences from *Vitis vinifera*, *Solanum lycopersicum *** **and *Arabidopsis thaliana*.** Click here for file

Additional file 7 List of primer sequences used in RT-PCR analysis. Click here for file
