# Supplementary material for: The onset of grapevine berry ripening is characterized by ROS accumulation and lipoxygenase-mediated membrane peroxidation in the skin
Source: BMC Plant Biol. 2014 Apr 2;14:87. doi: 10.1186/1471-2229-14-87 (PMC4021102; doi:10.1186/1471-2229-14-87)
Supplement: Additional file 1 — H 2 O 2 content quantification and lipid peroxidation analysis of Pinot noir berry skin extracts during season 2008. [file 1471-2229-14-87-S1.pdf]

"The onset of grapevine berry ripening is characterized by ROS accumulation and lipoxygenase-mediated membrane peroxidation in the skin"

by Pilati S, Brazzale D, Guella G, Milli A, Ruberti C, Biasioli F, Zottini M and Moser C

A

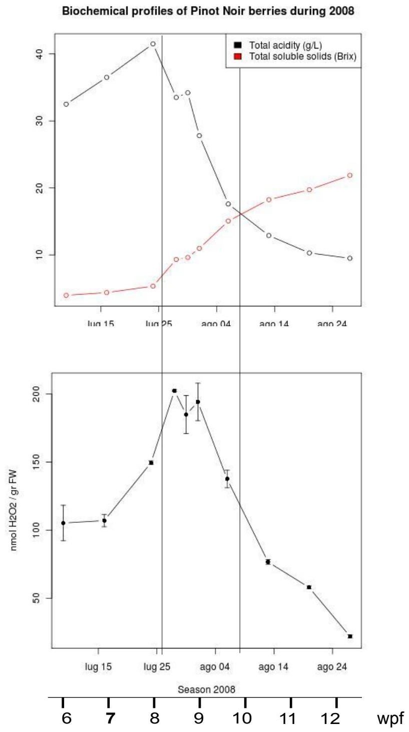

Additional file 1A: Biochemical profiles and H_2O_2 content in Pinot Noir grape berry skin during 2008. The acidic and soluble solids profiles allow to set véraison (interval indicated by the two vertical lines). H_2O_2 content in skin samples has been assayed with Amplex Red (Molecular Probes). *n*=3, bars represent standard error.

B

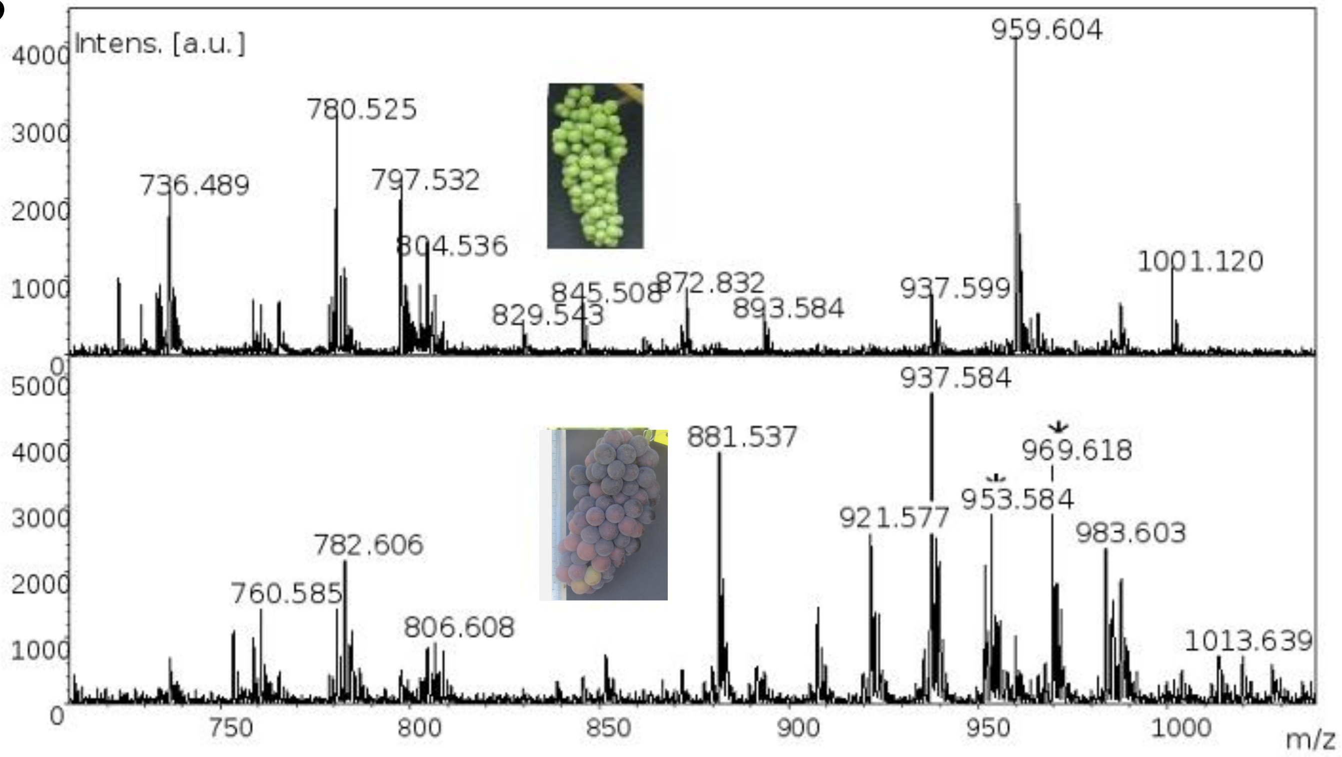

Additional file 1B: Galactolipid peroxidation in Pinot Noir grape berry skin sampled during 2008 analyzed by positive MALDI-TOF. Spectra of raw lipid extracts of green hard berry skin samples at 7 wpf (July, 16th, top) and berry skin samples at véraison, 9 wpf (August, 1st, bottom) are shown. The ions at the nominal m/z 953 and 969 (*) in the véraison sample have been assigned as [M+H+O]+ and [M+H+2O]+ of the corresponding [M+H]+ ion at m/z 937 in the green hard berry sample, which has been attributed to native DGDG 18:3/18:3.
