# Supplementary material for: The onset of grapevine berry ripening is characterized by ROS accumulation and lipoxygenase-mediated membrane peroxidation in the skin
Source: BMC Plant Biol. 2014 Apr 2;14:87. doi: 10.1186/1471-2229-14-87 (PMC4021102; doi:10.1186/1471-2229-14-87)
Supplement: Additional file 3 — Updated description of the Vitis vinifera LOX gene family; heatmap of the LOX gene family expression in the V. vinifera cv Corvina atlas and transcriptional profiles of the 5 LOX genes expressed in Corvinia berries. [file 1471-2229-14-87-S3.pdf]

Update of the LOX family described in Podolyan et al, 2011 to the new genome annotation (12X, V1)

| ID (Podolyan et al., 2011) | Gene name (12X, V1 annotation) | Chromosome (strand) | Predicted protein length | Predicted functionality |
|---|---|---|---|---|
| VvLOXA | Vv06s0004g01510 | Chr 6(-) | 901 | chlor 13-LOX |
| VvLOXB | Vv14s0128g00790 | Chr 14(+) | 869 | 9-LOX |
| VvLOXC | Vv14s0128g00780 | Chr 14(+) | 859 | 9-LOX |
| VvLOXE | Vv06s0004g01450 | Chr 6(+) | 859 | 13-LOX |
| VvLOXF | Vv06s0004g01460 | Chr 6(+) | 598 | 13-LOX |
| VvLOXG | Vv06s0004g01470 | Chr 6(+) | 903 | 13-LOX |
| VvLOXH | Vv06s0004g01480 | Chr 6(+) | 793 | 13-LOX |
| VvLOXI | Vv06s0004g01500 | Chr 6(+) | 791 | 13-LOX |
| VvLOXJ | Vv13s0064g01480 | Chr 13(+) | 903 | 13-LOX |
| VvLOXK | Vv13s0064g01490+Vv13s0064g01500 | Chr 13(+) | 399+ 250 | Pseudogene? |
| VvLOXL | Vv05s0020g03170 | Chr 5(+) | 876 | 9-LOX |
| VvLOXM | Vv06s0004g01580 | Chr 6(-) | 335 | Pseudogene? |
| VvLOXO | Vv09s0002g01080 | Chr 9(+) | 927 | 13-LOX |
| VvLOXP | Vv01s0010g02750 | Chr 1(-) | 920 | 13-LOX |
| VvLOXS | Vv00s0265g00170 | Chr Un (-) | 505 | 13-LOX |

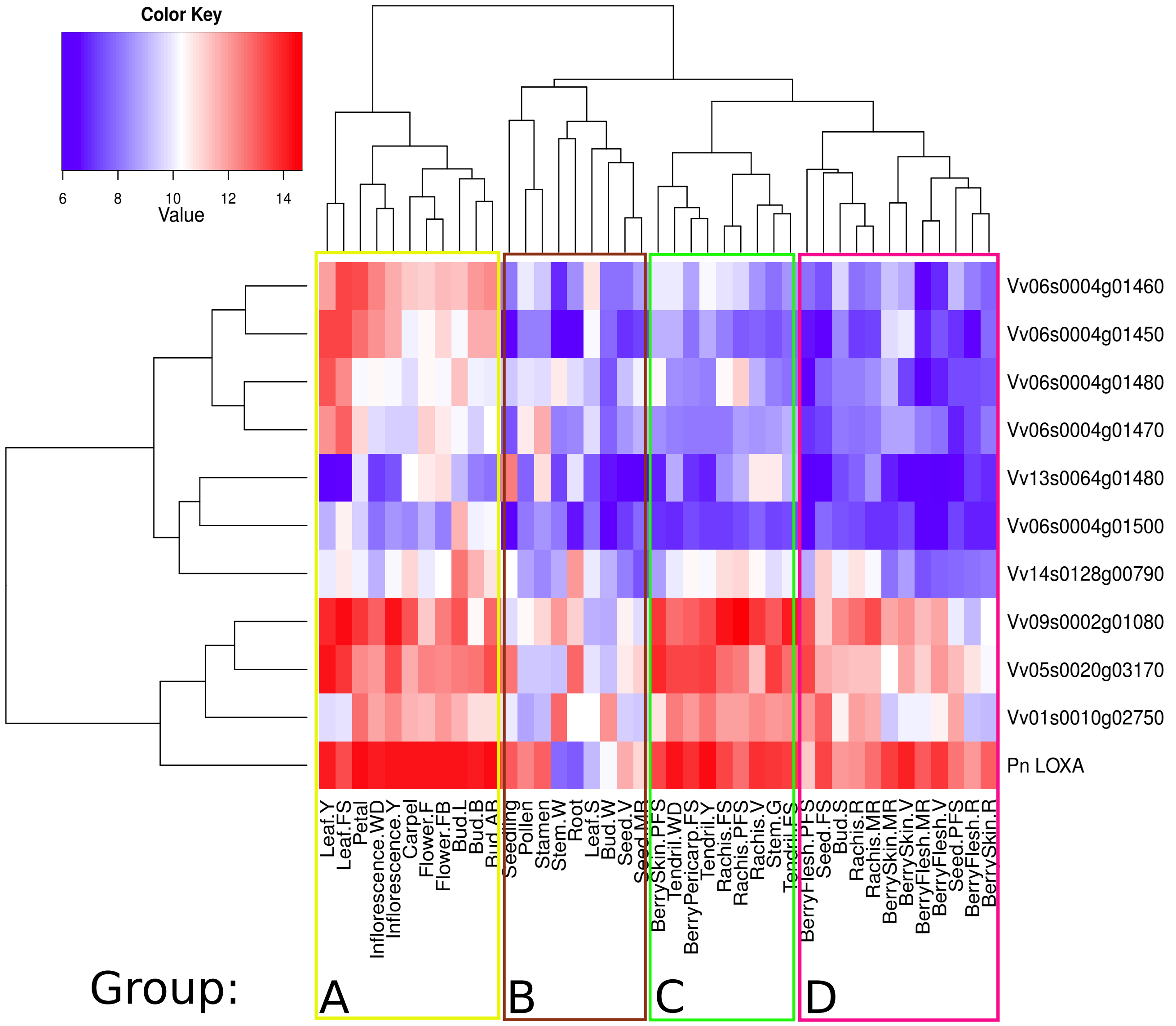


**Additional file 3A: Updated description of the *Vitis vinifera* LOX gene family described by Podolyan et al. (2012) and heatmap of the LOX gene family expression in the *V. vinifera* cv Corvina atlas (Fasoli et al., 2012).** The lox sequences identified by Podolyan et al. (2012) have been updated with the gene ID of the V. vinifera genome 12Xv1 (Grimplet J et al., 2012 BMC Res Notes 5:213). In the heatmap, colors correspond to expression values as indicated in the scheme at top left. The most interesting organs/tissues were selected and reported on the columns. Predicted LOX genes (and PnLOXA) are shown on the right. VvLOXB and VvLOXC, on Chr 14, are 96% identical and the probes on the Nimblegene array are not specific enough to distinguish them. VvLOXS was not expressed in any sample and was therefore excluded from the heatmap analysis.
Y: young; WD: well developed; Bud-L: latent bud; Bud-W: winter bud; Bud-S: bud swell; Bud-B: bud burst; Bud-AB: bud after burst; Flower-FB: flowering begins; Flower-F: flowering; S: senescent; FS: fruit set; PFS: post-fruit set; V: véraison; MR: mid-ripening; R: ripening; Stem-G: green stem; Stem-W: woody stem (Fasoli et al., 2012).
Group A: samples in which nearly all the LOX genes are expressed (leaf, bud and inflorescence). Group B: samples in which there is a very LOX isoform-specific expression (non-photosynthetic tissues). Groups C and D: samples in which five LOX isoforms are significantly expressed and modulated (fruit, rachis and tendril).

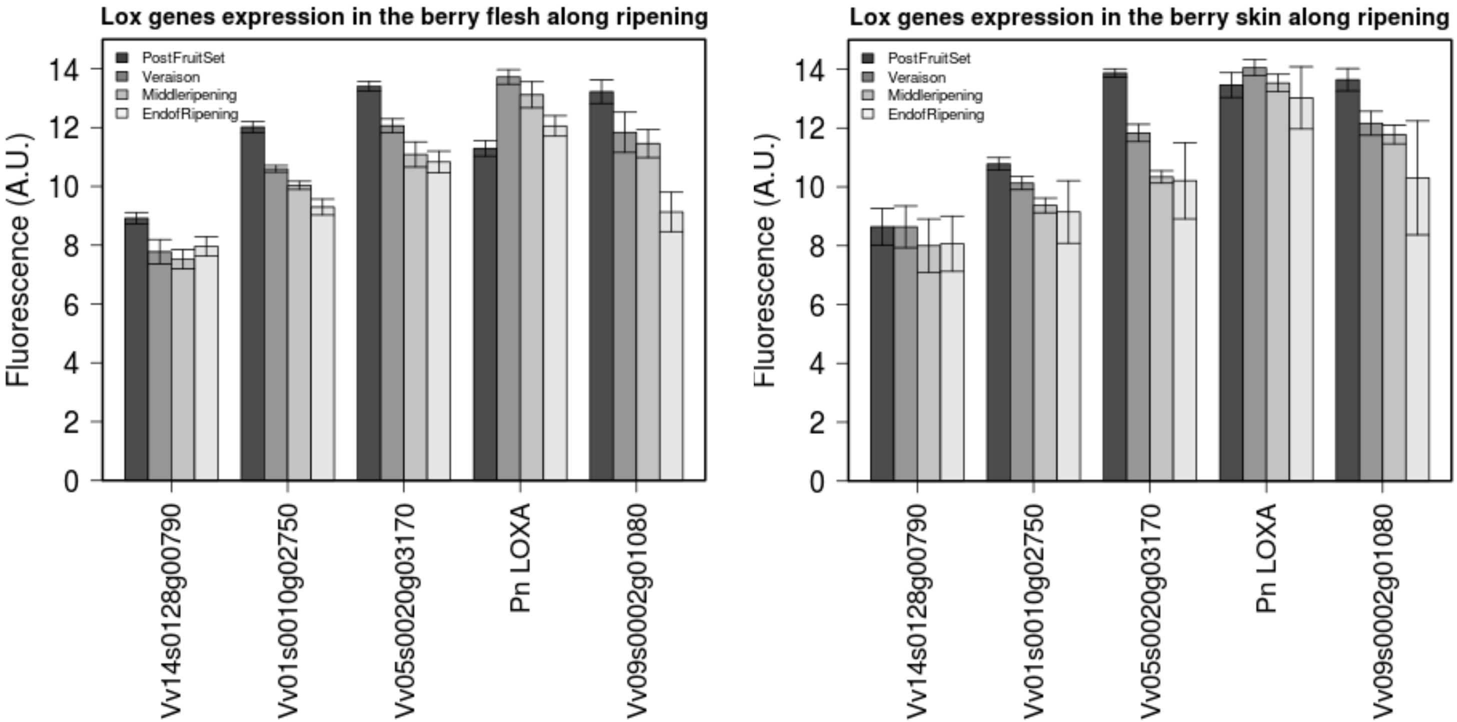


**Additional file 3B: Expression profiles of the 5 LOX isoforms expressed in the berry along ripening reported for *V. vinifera* cv Corvina in the atlas by Fasoli et al., 2012.** The fluorescence average value calculated on the probesets of each gene in the microarray experiments is reported on the y-axis. Bars represent the standard error. PnLOXA is the only isoform showing a specific induction at véraison stage.
