# Supplementary material for: The onset of grapevine berry ripening is characterized by ROS accumulation and lipoxygenase-mediated membrane peroxidation in the skin
Source: BMC Plant Biol. 2014 Apr 2;14:87. doi: 10.1186/1471-2229-14-87 (PMC4021102; doi:10.1186/1471-2229-14-87)
Supplement: Additional file 4 — PnLOXA localization demonstrated by transient expression of YFP fusion constructs in tobacco leaves. [file 1471-2229-14-87-S4.pdf]

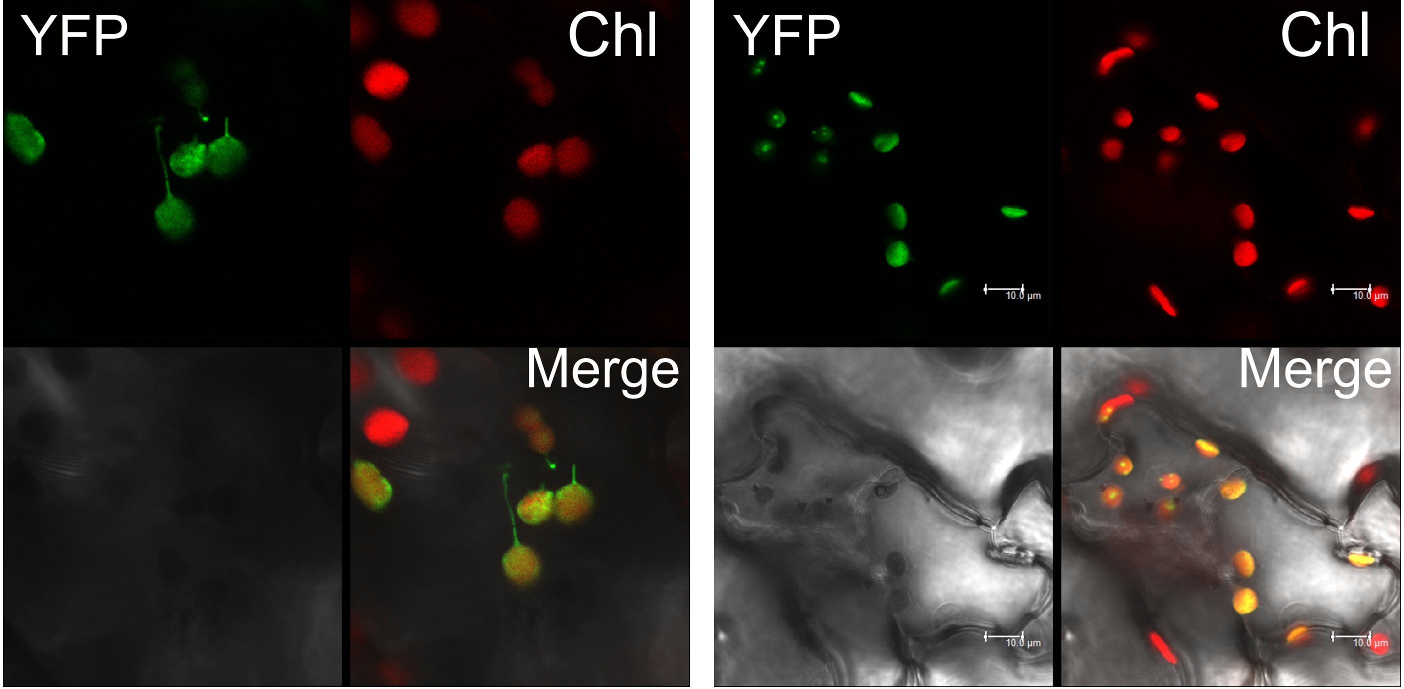


**Additional file 4: PnLOXA localization demonstrated by transient expression of YFP fusion constructs in tobacco leaves.** Tobacco leaves were infiltrated with *Agrobacterium tumefaciens* carrying the two constructs pGreen[PnLOXAtransitpeptide1-47-YFP] (left panel) and pGreen[PnLOXAtransitpeptidePLAT1-220-YFP] (right panel). Chlorophyll and YFP fluorescence were recorded using Leica SP II confocal microscope. Reference bar is 10 micron.
