# Supplementary material for: The onset of grapevine berry ripening is characterized by ROS accumulation and lipoxygenase-mediated membrane peroxidation in the skin
Source: BMC Plant Biol. 2014 Apr 2;14:87. doi: 10.1186/1471-2229-14-87 (PMC4021102; doi:10.1186/1471-2229-14-87)
Supplement: Additional file 5 — Western analysis of tobacco leaves transiently overexpressing PnLOXA . [file 1471-2229-14-87-S5.pdf]

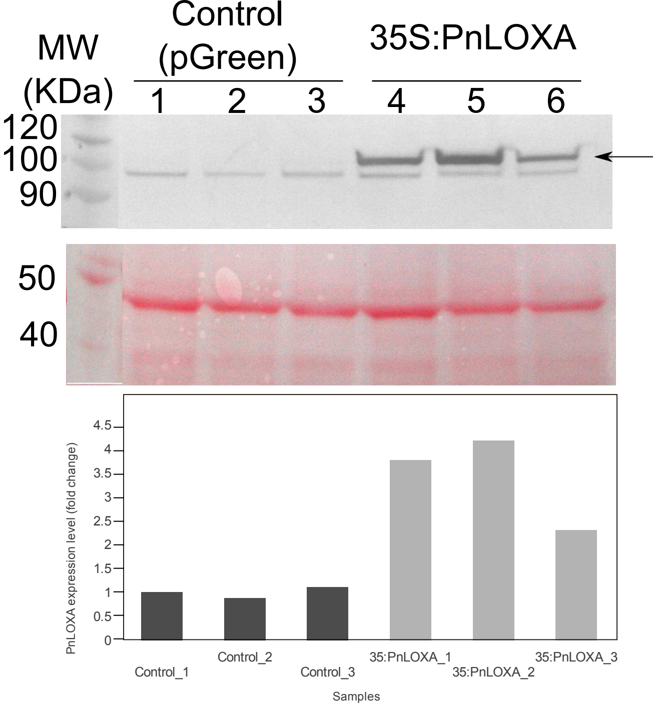

Additional file 5: Expression analysis of PnLOXA in tobacco leaves transiently transformed with either empty pGreen (control) or pGreen[35S:*PnLOXA*]. Six tobacco plants were infiltrated with Agrobacterium carrying the two plasmids and transformed leaves were sampled after 7 days. Western blot analysis of total protein extracts was performed to assay the overexpression of the grapevine gene. Ponceau and western blot images were scanned and analyzed with ImageJ software to calculate an expression fold change, used to normalize peroxidized galactolipids measured by HPLC-MS.
