# Supplementary material for: The onset of grapevine berry ripening is characterized by ROS accumulation and lipoxygenase-mediated membrane peroxidation in the skin
Source: BMC Plant Biol. 2014 Apr 2;14:87. doi: 10.1186/1471-2229-14-87 (PMC4021102; doi:10.1186/1471-2229-14-87)
Supplement: Additional file 6 — Phylogenetic analysis of LOX sequences from Vitis vinifera , Solanum lycopersicum and Arabidopsis thaliana . [file 1471-2229-14-87-S6.pdf]

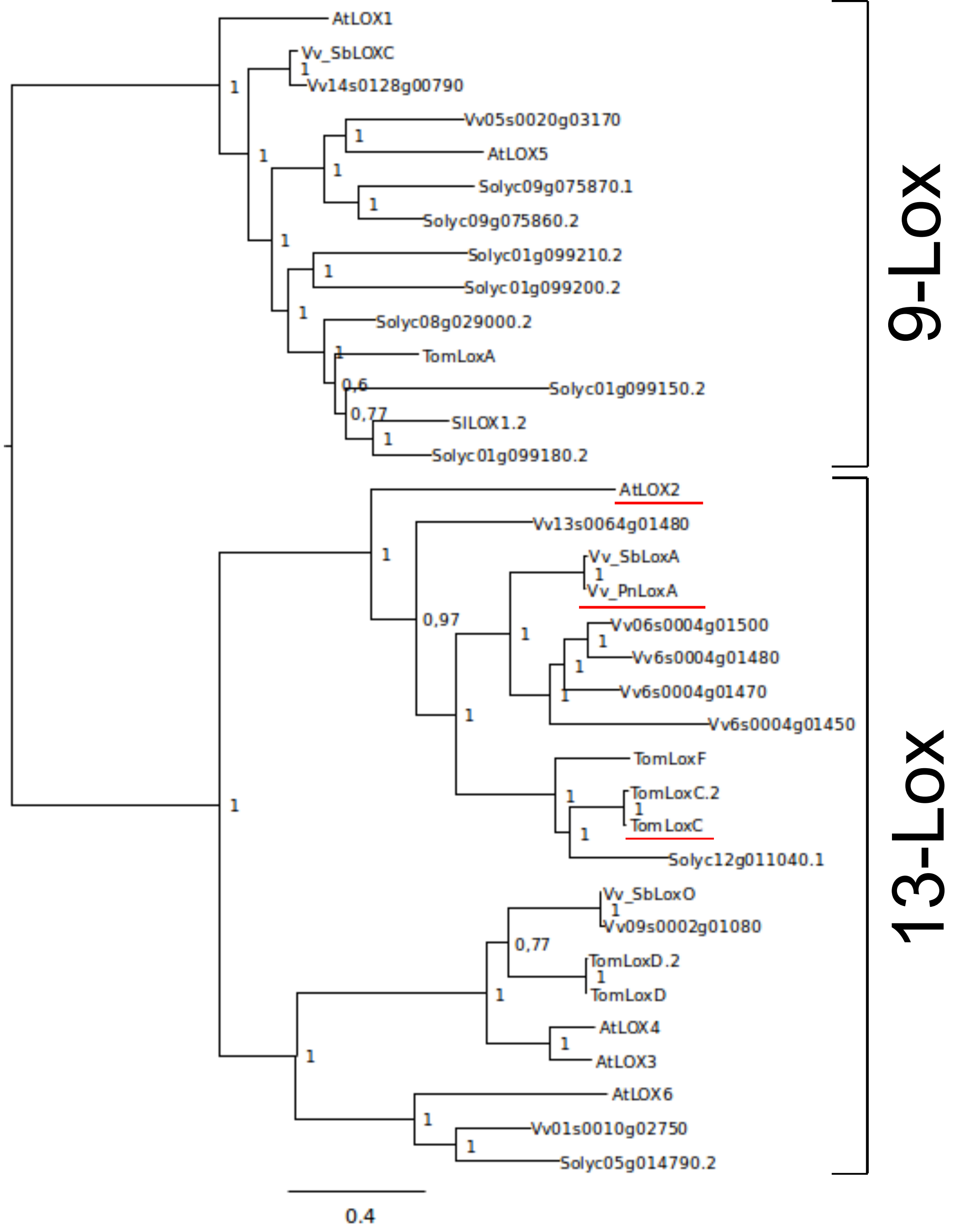

Additional file 6: Phylogenetic analysis of *Vitis vinifera, Solanum lycopersicum* and *Arabidopsis thaliana* Lox families.
Bayesian tree using PhyloBayes3 (Lartillot et al. 2009) and the LG+Gamma model (Le and Gascuel 2008).
Support at nodes are posterior probabilities from the consensus of two independent, satisfactorily converged Bayesian analyses (maxdiff statistics lower than 0.3). The alignment was done using Muscle (http://www.ebi.ac.uk/Tools/msa/muscle/help/).
The Uniprot ID of the sequences analyzed are listed in brackets: A. thaliana sequences: AtLOX1 (Q06327), AtLOX2 (P38418), AtLOX3 (Q9LNR3), AtLOX4 (Q9FNX8), AtLOX5 (Q9LUW0), AtLOX6 (Q9CAG3); V. vinifera sequences: PnLOXA (Vv06s0004g01510, D7SLA9), SbLOXA (D5FUD8), Vv06s0004g01450 (F6GUD0), Vv06s0004g01470 (F6GUA7), Vv06s0004g01480 (F6GUA6), Vv06s0004g01500 (F6GUA5), Vv13s0064g01480 (F6HB91), Vv14s0128g00790 (F6HZ11), Vv01s0010g02750 (D7TAQ3), Vv09s0002g01080 (D7TZR1), SbLOXO (D5FUE0), SbLOXC (D5FUD9), Vv05s0020g03170 (F6HDN6); Solanum lycopersicum sequences: TomLoxA (P38415), TomLoxB (P38416), TomLoxC (K4ASM0), TomLoxC.2 (Q96573), TomLoxD (K4BN32), TomLoxD.2 (Q96574), TomLoxF (C0KKU8), Solyc12g011040.1 (K4DCJ0), Solyc01g099150.2 (K4B0V2), Solyc05g014790.2 (K4BYJ7), Solyc01g099200.2 (K4B0V7), Solyc09g075860.2 (K4CVG3), Solyc01g099210.2 (K4B0V8), Solyc08g029000.2 (K4CJW3), Solyc09g075870.1 (K4CVG4), Solyc01g099180.2 (K4B0V5).
